# Randomized clinical trials to identify optimal antibiotic treatment duration

**DOI:** 10.1186/1745-6215-14-88

**Published:** 2013-03-28

**Authors:** C Robert Horsburgh, Kimberly M Shea, Patrick Phillips, Michael LaValley

**Affiliations:** 1 Department of Epidemiology, Boston University School of Public Health, 715 Albany Street, Boston, MA, 02118, USA; 2 Department of Biostatistics, Boston University School of Public Health, 801 Massachusetts Avenue, Boston, MA, 02118, USA; 3 Department of Medicine, Boston University School of Medicine, 715 Albany Street, Boston, MA, 02118, USA; 4 MRC Clinical Trials Unit, Aviation House, 125 Kingsway, London, WC2B 6NH, UK

**Keywords:** Antibiotic trials, Antibiotic treatment duration, Tuberculosis, Logistic regression

## Abstract

**Background:**

Antibiotic resistance is a major barrier to the continued success of antibiotic treatment. Such resistance is often generated by overly long durations of antibiotic treatment. A barrier to identifying the shortest effective treatment duration is the cost of the sequence of clinical trials needed to determine shortest optimal duration. We propose a new method to identify the optimal treatment duration of an antibiotic treatment regimen.

**Methods:**

Subjects are randomized to varying treatment durations and the cure proportions of these durations are linked using a logistic regression model, making effective use of information across all treatment duration groups. In this paper, Monte Carlo simulation is used to evaluate performance of such a model.

**Results:**

Using a hypothetical dataset, the logistic regression model is seen to provide increased precision in defining the point estimate and confidence interval (CI) of the cure proportion at each treatment duration. When applied to the determination of non-inferiority, the regression model allows identification of the shortest duration meeting the predefined non-inferiority margin.

**Conclusions:**

This analytic strategy represents a practical way to develop shortened regimens for tuberculosis and other infectious diseases. Application of this strategy to clinical trials of antibiotic therapy could facilitate decreased antibiotic usage, reduce cost, minimize toxicity, and decrease the emergence of antibiotic resistance.

## Background

Antibiotic therapy for the treatment of infectious diseases transformed the practice of medicine in the twentieth century. However, as that century drew to a close, antibiotic resistance emerged as a major barrier to the continued success of antibiotic treatment [1]. Since then, we have learned that antibiotic resistance has many predisposing factors, including selective pressure of non-curative regimens, genetic exchange of resistance mutations between microbial species and indiscriminate use of antibiotics for non-bacterial conditions [2].

Identification of antibiotic regimens that minimize the emergence of resistance is hampered by limited information about the optimal duration of antibiotic treatment able to cure disease without causing microbial resistance. While pharmacokinetic parameters are used to identify antibiotic doses that provide the most advantageous balance between efficacy and tolerability, few antibiotic regimens have been subjected to rigorous evaluation of treatment duration. There are notable exceptions, of course; the appropriate durations of antibiotic treatment necessary for urinary tract infections in women and for sexually transmitted diseases have been well studied [3], and a large series of trials established the shortest effective antibiotic treatment regimen for tuberculosis [4]. However, data are lacking for the optimal treatment duration for many other diseases, including diarrhea, meningitis and pneumonia. Moreover, the likely introduction of new antibiotics for the treatment of tuberculosis in the coming decade will require re-evaluation of the shortest optimal tuberculosis treatment duration [5,6].

Since treatment for too short a period of time can lead to unacceptably high failure proportions, pre-licensure drug studies usually target a period of time that is somewhat longer than the likely minimum. Once an initial effective therapy has been established, subsequent trials to assess the effectiveness of shorter durations of the same regimen, or new regimens to treat the same condition, are evaluated in equivalence trials designed to show that the new duration or regimen can achieve a cure proportion that is within a small ‘margin of inferiority’ of the cure proportion of the accepted standard regimen [7]. This means that in order for a new duration or antibiotic to be approved for use, there must be sufficient evidence that it is no less effective than the standard regimen by more than a pre-specified amount. Trials that seek to show ‘non-inferiority’ require, of necessity, large numbers of subjects (*N*) because the required width of the 95% confidence interval (CI) around the point estimate is often the determining factor. Thus, they are very costly.

The result is that there is strong pressure to conduct studies or trials that evaluate only one treatment duration, since evaluation of multiple durations (*d*) would require separate study arms for each time period and a total of *N* × *d* study subjects. To minimize the risk of failing to demonstrate non-inferiority, a single treatment duration that is substantially greater than the theoretical minimum duration required is often selected and these study results are used to establish treatment guidelines. The outcome is that the shortest optimal duration of treatment is almost never identified and we are left in the unenviable position of prescribing antibiotics for longer durations than are likely necessary [3]. The downside of this approach, as clearly identified by Rice [3], is that every additional day of antibiotic treatment beyond the minimum required to cure disease increases the risk that host flora will develop resistance to the antimicrobial agent, not to mention additional costs to patients and health providers. Therefore, it behooves us to determine the minimum duration of antibiotic treatment of any given infectious disease. An additional incentive to determine the minimum duration of treatment is that for diseases where the duration is substantial, such as tuberculosis, reducing the duration of treatment can substantially reduce cost and increase adherence [8].

In this paper, we propose use of an alternative clinical trial design to evaluate varying durations of antibiotic regimens for the treatment of infectious diseases within a single trial. This trial design should be particularly useful when the standard of treatment is highly effective and the expected number of treatment failures is small because non-inferiority designs are usually employed for these types of studies. The design that we propose does not reduce the overall sample size necessary to conduct a trial compared to a non-inferiority trial of a single experimental regimen, but takes advantage of the relationship between gradually increasing durations to allow identification of the optimal treatment duration, with modest increase in sample size compared to the traditional evaluation of a single treatment arm.

## Methods

We describe a trial design to evaluate several treatment durations simultaneously, thereby facilitating selection of the treatment duration that best satisfies a set of pre-specified criteria. While this could be accomplished by conducting a large clinical trial that includes multiple trial arms of varying treatment durations where each arm is sufficiently powered to produce a stable estimate of the cure proportion for each duration, such an approach would be cost and time prohibitive. Instead, we propose using a logistic regression framework to link the cure proportions from several smaller trial arms of varying treatment durations in order to make effective use of information across all treatment duration groups [9], allowing for the evaluation of several trial arms of varying treatment duration without having to power the study for each individual trial arm.

Our proposed approach utilizes a postulated connection between increasing durations of treatment and subsequent increases in the cure proportion. In this paper, we will assume a linear relationship between the log odds of the cure proportion and the duration of treatment. We believe this is a reasonable, although approximate, relationship to assume for antibiotic treatment. While this is a convenient form for use in logistic regression modeling, other non-linear relationships could be also considered. The form of the relationship between duration and the outcome could vary by the medical condition and treatment under study, and should be considered in planning for such a trial.

Our goal differs from the objective of a more traditional trial design approach in that we do not wish to compare a single new treatment duration to an existing standard treatment. Instead, we wish to evaluate the effects of several durations of treatment (*d*_1_*, d*_2_*, … d*_s_) on the cure proportion *C*. We propose selection of varying treatment durations that range from the shortest to the longest duration that provides clinical equipoise. The total study sample (*N*) would then be randomized into strata (s) equal to the number of treatment durations, and each stratum would then have *N*_1_ = *N*_2_ = *N*_s_ number of subjects. Thus, the trial would yield s proportions, where *C*_1,_*C*_2,_*… C*_s_ represent the cure proportions in each individual stratum associated with that stratum-specific duration of treatment *d*_1_ to *d*_s_.

Dividing the population into smaller groups yields an opportunity to gather information about different treatment durations simultaneously. However, the cost of this opportunity is the loss of some of the statistical precision of the cure proportion observed in each stratum, resulting in larger CIs around the point estimates obtained within each individual stratum compared to what would have been achieved if all subjects had been in a single stratum and allocated to a single treatment duration. We propose that this loss of statistical precision can be mitigated by utilizing the relationship between treatment duration and cure proportion in a statistical model, thus giving information about the relationship between *C*_*i*_ and *d*_*i*_ for a range of *i* rather than just estimating *C*_*i*_ for one duration *d*_*i*_. The following logistic regression model can be used to relate the duration of treatment to a cure proportion:

$$\log\left(\frac{P_{\mathrm{ij}}}{1-P_{\mathrm{ij}}}\right)=\alpha +\gamma d_{i}$$

where *P*_*ij*_ is the probability of a cure in patient *j* in duration group *i*, and *d*_*i*_ is the duration of treatment for duration group *i*. The parameters in this logistic regression model can be related to various quantities of interest for the trial. The parameter *α* corresponds to the log odds of a cure without treatment, and the parameter *γ* corresponds to the log odds ratio for a cure corresponding to an increase in the duration of the new treatment by one unit. While this logistic regression model uses the postulated linear relationship between treatment duration and the log odds of a cure, the model could be changed to reflect other forms for this relationship as needed.

## Results

### Estimating cure proportion

**Figure 1:**
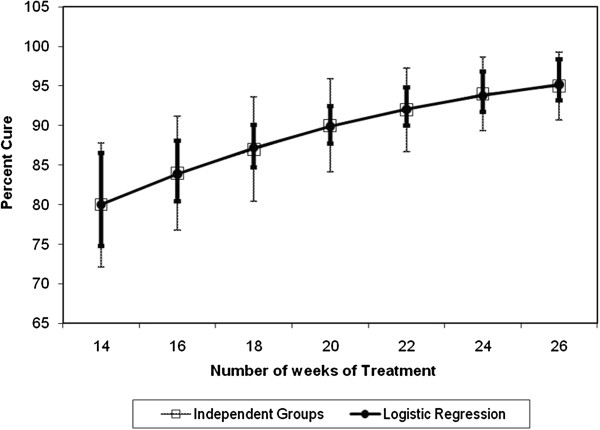
**Comparison of cure proportions and 95% CIs obtained from conventional trial design with 100 subjects per arm and logistic regression model of proposed trial design with 100 subjects per arm.** The number of weeks of treatment is shown on the x-axis, while the cure proportion is shown on the y-axis. Open boxes and light bars show the point estimate and 95% CI using independent samples, while the closed circles and dark bars show the point estimates and 95% CIs using the proposed regression model and the same number of study subjects as in the independent samples. CI, confidence interval.

Estimates derived from fitting this logistic regression model to the hypothetical trial data allow the calculation of the predicted probability (and a 95% CI around this probability) of a cure for selected treatment durations taking into account the estimate of the cure proportion at each of the other treatment durations included in the model. The statistical advantage of this modeling approach is that although the sample size in each individual stratum has been made smaller, the estimate of the cure proportion will be made more precise by using information from the other strata, assuming that the specified model is acceptable. Figure 1 demonstrates this by showing: a. the cure proportion and 95% CI for a hypothetical sample of 100 subjects calculated for each of seven selected treatment durations without consideration of the other strata; and b. the cure proportion and 95% CI for a hypothetical sample of 100 subjects calculated for each of seven treatment durations including the other strata using logistic regression as described above.

### Use of the logistic regression model in conjunction with a control arm to establish non-inferiority

Standard treatment regimens already exist for many diseases. We therefore propose an extension of our approach to include comparison of multiple durations of treatment to a ‘standard’ therapeutic regimen using the following logistic regression model:

$$\log\left(\frac{P_{\mathrm{ij}}}{1-P_{\mathrm{ij}}}\right)=\alpha +\left(\beta +\gamma d_{i}\right)z_{\mathrm{ij}}$$

where *P*_*ij*_ is the probability of a cure in patient *j* in duration group *i*, *d*_*i*_ is the centered duration of treatment for duration group *i*, and *z*_ij_ is an indicator term for type of treatment; the treatment indicator will be equal to 0 for subjects who receive the standard regimen and equal to 1 for subjects who receive any duration of the new regimen. The parameter *α* corresponds to the log odds of a cure on the standard regimen. The parameter *β* is the log odds ratio comparing the cure proportion of the new regimen when *d*_*i*_ is equal to 0 to that of the standard regimen. We center the values of *d*_*i*_ at 0 to make *β* a useful quantity to estimate, which would otherwise be a necessary, but not interpretable, intercept. Finally, the parameter *γ* corresponds to the log odds ratio for a cure corresponding to an increase in the duration of the new regimen by one unit. In this model, the odds ratio of a cure comparing any duration group to the standard regimen is exp(*β* + *γd*_*i*_) where *d*_*i*_ is the centered duration value for the group. As above, this logistic regression model could be changed to reflect non-linear relationships between treatment durations and the log odds of a cure.

### Simulation studies

The following is an example of the application of our proposed trial design to a hypothetical clinical trial of non-inferiority whose aim is to identify the shortest duration for tuberculosis antibiotic therapy using new Regimen A that is not inferior to treatment with the current standard regimen. We hypothesize, based on results obtained from animal models and the observed efficacy of Regimen A of killing *Mycobacterium tuberculosis in vitro*, that durations as short as 14 weeks and as long as 26 weeks could achieve the desired cure proportion, and thus the shortest effective regimen would lie somewhere between these two durations.

A conventional trial might allocate 700 subjects to a standard regimen and 700 subjects to 20 weeks of the experimental treatment, Regimen A. In the proposed trial design, instead of allocating all 700 subjects who receive Regimen A to a single treatment duration, we elect to distribute the 700 subjects as follows: 100 subjects each will receive treatment with Regimen A for 14, 16, 18, 20, 22, 24 and 26 weeks. To compare this to the conventional trial design, we use simulated data to see how the two designs perform under repeated realizations of the data that might be generated under ideal conditions. In particular, we focus on the proportion of simulated trials under each design that are able to successfully determine the non-inferiority of Regimen A at 20 weeks’ duration to the standard regimen.

To simulate the data for these ideal trials, we use the proposed logistic regression model including a standard regimen arm with the following parameter values: *α* = 2.2, *β* = 0 and *γ* = 0.136. These values were chosen to provide the cure proportions listed in Table 1. The data for 10,000 trials were randomly generated for both the conventional and proposed trial designs.

**Table 1:** Cure proportions for 10,000 simulated trials using conventional and proposed trial designs

**Trial design and treatment**	**Number of subjects**	**Treatment duration (weeks)**	**Cure proportion**
*Conventional trial design*			
Standard regimen	700	NA	0.90
Regimen A	700	14	0.90
*Proposed alternative trial design*			
Standard regimen	700	NA	0.90
Regimen A	100	14	0.80
Regimen A	100	16	0.84
Regimen A	100	18	0.87
Regimen A	100	20	0.90
Regimen A	100	22	0.92
Regimen A	100	24	0.94
Regimen A	100	26	0.95

To determine the non-inferiority boundary on the odds scale, we first considered a possible non-inferiority margin for the difference in proportions. If the cure proportion in the control regimen was 0.90, one might consider a cure proportion of 0.85 to be reasonable for a shorter treatment regimen, resulting in a non-inferiority margin of the difference of the cure proportions equal to 0.05. This corresponds to an odds ratio of 0.63 as the margin of non-inferiority. For this example, non-inferiority would be demonstrated if the lower bound of the 95% CI around the odds ratio estimate comparing a given duration of Regimen A to the standard regimen is greater than 0.63.

**Figure 2:**
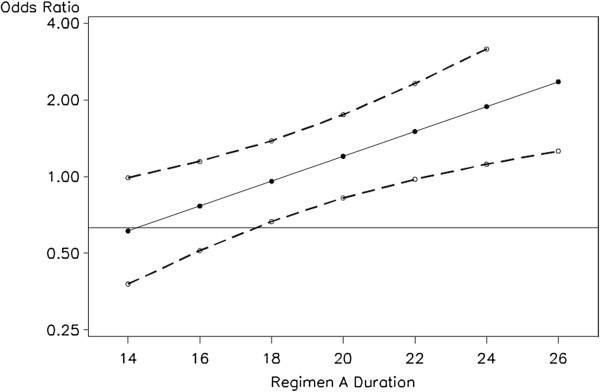
**Logistic regression estimates of odds ratios for a cure on Regimen A compared to standard regimen by duration of treatment with Regimen A in a single simulated trial using the proposed trial design.** Odds ratio estimates are shown by the solid circles connected with the solid line, while the upper and lower 95% CI bounds for these estimates are shown with solid circles connected by dashed lines. The non-inferiority boundary of 0.63 is shown by the horizontal line at that level on the vertical axis. The vertical axis is presented on the log scale. CI, confidence interval.

To provide odds ratio estimates and 95% CIs for the simulated trials we used the LOGISTIC Procedure in SAS (SAS Institute, Cary, NC, USA). In analyses of the proposed trial design the centered duration (*d*_*i*_) was included as a linear predictor, allowing the proportion of Regimen A subjects responding at each of the seven durations to influence the width of the CI for all of the odds ratios comparing Regimen A to the standard regimen. Subjects on the standard regimen had their value of *d*_*i*_ set to 0, and there was no duration included in the logistic regression model for the standard trial design. Figure 2 shows the results of applying the proposed logistic regression model to data generated in a single simulated trial using the proposed trial design. The odds ratios comparing each treatment duration of Regimen A to the standard regimen are plotted along with upper and lower 95% CI bounds. At 20 weeks’ duration, Regimen A would be considered to be non-inferior to the standard regimen as the lower bound of the CI is above the non-inferiority margin of 0.63. In fact, in this particular simulated dataset, all Regimen A treatment durations of 18 weeks or longer would be judged to be non-inferior to the control.

The results from the simulations are shown in Table 2. This table gives average values across the 10,000 simulations of the predicted cure proportions, log odds of a cure, and standard error of the log odds for both the conventional and proposed trial designs by treatment and duration groups. We see that the standard error of the predicted log odds values for 20 weeks’ duration of treatment on Regimen A in the proposed trial is close to the standard error of the log odds for Regimen A subjects in the conventional trial, despite the fact that in the conventional trial all 700 subjects on Regimen A are treated for 20 weeks and only 100 subjects receive Regimen A for exactly 20 weeks in the proposed alternative trial design; the subjects treated for other durations in the proposed trial design do provide precision for the predicted probability (and log odds) of a cure at 20 weeks. Table 3 presents the proportion of simulations where non-inferiority was reached in comparing the 20 week duration of Regimen A to the standard treatment. The proportion of simulated trials that achieve non-inferiority is 73.4% in the conventional trial design, which is approximately the same as in the proposed alternative trial design, 72.4% (*P* = 0.62, χ^2^ test for independence). However, the proposed design provides substantial additional information about other durations of treatment on Regimen A.

**Table 2:** Predicted cure proportion, log odds and standard error of the log odds by trial type, treatment regimen and duration (average values across the 10,000 simulations)

**Trial type and regimen**	**Duration in weeks**	**Average cure proportion**	**Average log odds of cure**	**Standard deviation of log odds of cure**
*Conventional trial design*				
Standard regimen		0.900	2.206	0.128
Regimen A	20	0.900	2.207	0.128
*Proposed trial design*				
Standard regimen		0.900	2.207	0.128
Regimen A	14	0.799	1.390	0.190
Regimen A	16	0.840	1.665	0.146
Regimen A	18	0.874	1.939	0.125
Regimen A	20	0.901	2.214	0.136
Regimen A	22	0.922	2.488	0.174
Regimen A	24	0.939	2.763	0.225
Regimen A	26	0.953	3.037	0.283

To investigate the role of linearity of duration effect on the design efficiency, we applied the same logistic model to data simulated so that the effect of duration on the log odds of a cure was not linear. To keep the range of cure proportions reasonable, the simulations were set so that the cure proportions matched those of the previous at the extremes provided by weeks 14 and 26 (Table 1). In addition, to prevent biasing the comparisons, the cure rate at week 20 was kept the same as for the standard treatment and the symmetry in the log odds around week 20 was maintained. Log odds values for these new simulations were generated using step functions, and cubic and cube-root polynomials. The simulation using a step function placing most of the log odds at the extreme values (cure proportions of 0.8 for all durations less than 20 weeks and cure proportions of 0.95 for all durations longer than 20 weeks) provided the worst results for the logistic regression model. In this simulation non-inferiority for the 20 week duration was attained in 64% of the datasets as opposed to the 72.4% when data were simulated as in Table 1. The other simulations with non-linear log odds values had non-inferiority at week 20 attained for between 69% and 72% of the simulated data sets.

It should be noted that equal allocation of subjects to the standard regimen group and the combined Regimen A treatment groups (as was done in these simulations) may not provide the most power to show non-inferiority. However, varying the allocation ratio between the standard arm and the seven experimental treatment duration arms did not provide greater power to reach non-inferiority. Varying the fraction of subjects allocated to the standard arm from 40% to 60% resulted in power values between 70.6% and 73.3% in such simulations. The standard arm proportion had to be substantially more extreme to reduce the power to a considerable extent: if 980 subjects (70%) were allocated to the standard arm and the remaining 420 subjects split into the seven treatment groups, power decreased to 65.5%, with similar results obtained from putting 30% of subjects into the standard arm. Although we have not pursued this, there may in some cases be benefit in considering the effect on power of varying the number of patients allocated to the different duration groups.

The proposed method of estimating a point estimate of the cure proportion and the CI around that estimate can be applied to clinical trials to determine non-inferiority of regimens of varying durations to an existing standard course of therapy. In the tuberculosis example, a standard arm consisting of the World Health Organization recommended 6-month tuberculosis treatment regimen would be compared to a single regimen of new drugs, given for several durations. The SAS code used for the power simulations in this section may be obtained from the authors by request.

### Application of proposed model to superiority comparisons

The proposed design could also be used with randomization to a placebo arm to evaluate new drugs for prevention of tuberculosis disease among infected contacts of persons with multiple-drug-resistant tuberculosis, since no effective treatment currently exists for this condition. Study of a single duration of a new agent that was relatively certain to be successful for this indication would mean that a superiority design could only be employed until a single effective regimen (likely longer than optimal) was identified. After this first successful single-duration trial, all subsequent trials would then need to be non-inferiority studies, greatly increasing the cost and time to identification of the optimal regimen. Instead, we propose studying smaller numbers of subjects randomized to several durations, so that the shortest duration that was superior to placebo could be determined.

## Discussion

In this paper, we used the example of tuberculosis to propose an alternative clinical trial design to evaluate varying durations of antibiotic treatment, with the objective of identifying the shortest effective treatment regimen. In the early days of tuberculosis chemotherapy, the Medical Research Council (UK) performed sequential clinical trials to identify the optimal duration of tuberculosis treatment. These trials succeeded in gradually shortening the duration of therapy from 18 to 6 months [4]. However, the process took place over a 30-year period and was complicated by the need to explain why the same regimen could have different performance characteristics in different geographic regions. Most of the trials were not powered to be definitive, but rather conclusions were reached when data accumulated over a long period of time became compelling. In the current situation, where large multi-site randomized clinical trials have the potential to enroll many patients and answer study questions expeditiously, such an iterative approach is not in the best interests of patients or the medical community. In addition, with several new anti-tuberculosis agents potentially becoming available for study within a few years of one another, the complexity of investigating different durations of each regimen could quickly exhaust both the number of patients and the resources available for such studies.

The proposed analytic strategy could also be applied to investigating the minimal duration of treatment of conditions where current treatment is prolonged and efficacious, but few shorter regimens have been examined, such as endocarditis and osteomyelitis. The impetus for such trials is likely to come from the need to minimize the cost of prolonged treatment, but there are additional benefits to be gained, such as reduction in unintended generation of resistance by continuation of antibiotics for longer durations than necessary. Such risks may be mediated by selection of antibiotic resistance in host organisms other than those that are the primary targets of the antibiotic course (such as commensal gut flora), and thus may be difficult to measure. In settings such as tuberculosis, where resistance is largely generated by antibiotic pressure, it may be possible to measure generation of antibiotic resistance directly, and this could be the primary endpoint of a clinical trial using the type of design outlined here.

The strategy that we describe is analogous to that used to optimize dose–response relationships in phase 1 and phase 2 clinical trials [10]. In such trials, several different doses are studied and the results are modeled to identify the dose that maximizes efficacy with acceptable toxicity. In the current study, we assess several different durations and model the results to identify the duration that maximizes efficacy without unnecessarily prolonging duration. To our knowledge, use of this type of modeling has not been previously described in analyzing results of phase 3 clinical trials. The example that we have given is simple and is meant to demonstrate the concept that simultaneous examination of multiple durations of a single regimen combined with multivariate logistic regression modeling will allow more efficient determination of the optimal duration of treatment. In addition, the results generated can be adjusted to account for measured confounders and imbalances in randomization.

The analytic approach that we have described has several limitations. First, there is a cost in precision associated with studying several durations simultaneously. However, unless there is strong a priori information to direct selection of the optimal treatment duration, study of a single duration carries substantial risk that the duration will be too brief, and thus not reach the cure target, or too long, establishing a regimen that exposes patients to excess antibiotic and increases the risk of emergence of antibiotic resistance. The method that we have proposed allows efficient use of patient populations to allow studies to determine the optimal duration of therapy without a series of sequential trials, each requiring its own control group. Moreover, there is minimal increase in sample size required to define the optimal duration, since information from each duration contributes to the optimization. Second, like any model-based approach, our analytic strategy could yield biased results if the relationship between duration and the outcome specified in the logistic regression model for the analysis did not reflect the true relationship. This implies that the relationship between duration and the outcome should be carefully considered prior to the trial, and should be pre-specified in the trial protocol. Since we expect to be examining durations that are near the optimally effective duration, we anticipate that the longest durations studied do not provide additional benefit, whereas the shortest ones studied will be slightly suboptimal. Thus, the slope of the regression line can be expected to be near the top of the duration-response curve and could reasonably be modeled as exponential, or linear on the log scale. Moreover, if this were the case, one could pre-specify use of a linear trend between the log of duration and the log odds of the outcome by replacing the duration *d*_*i*_ with the log of *d*_*i*_. Our simulations of possible scenarios where the duration effect was not linear revealed that the method still achieved interpretable results, albeit with some loss of power. Third, the logistic model estimates do depend on adequate counts of events in the different duration of treatment groups, so we would not recommend creating too many duration groups for the experimental treatment. Lastly, we were unable to find existing data from clinical trials that examined different durations of treatment with which to validate our proposed strategy. We invite anyone with access to such data to collaborate with us in such an effort.

## Conclusion

In conclusion, we propose that duration-randomized trials such as this be considered as a practical way to address the urgent need to develop shortened treatment regimens for tuberculosis. Using pairwise comparisons between many different duration groups can, with large enough samples, identify which of the durations employed in the study produced the highest proportion cured, but this approach does not allow one to say anything about durations not employed in the study. In contrast, the regression approach, at the price of some assumptions about the form of the effect of duration on proportion cured, allows us to directly estimate the shortest duration that would produce the best proportion cured, even if that exact duration were not included as one of the groups in the study. It also allows evaluation of the trade-offs between duration of treatment and the proportion of subjects cured. Application of this analytic strategy to clinical trials of antibiotic therapy for other infectious diseases could facilitate decreased antibiotic usage, thereby reducing cost, minimizing toxicity and lessening the risk of emergence of antibiotic resistance.

## Abbreviation

CI: Confidence interval

## Competing interests

The authors declare that they have no competing interests.

## Authors’ contributions

CRH conceived the manuscript, designed the study, participated in the data analysis and wrote the first draft of the manuscript. KMS performed some of the analyses and participated in writing the manuscript. PP participated in the study design, data analysis and writing the manuscript. ML performed some of the analyses, and participated in the data analysis and manuscript preparation. All authors read and approved the final manuscript.
